# Fine Mapping and Candidate Gene Analysis of *qSTL3*, a Stigma Length-Conditioning Locus in Rice (*Oryza sativa *L.)

**DOI:** 10.1371/journal.pone.0127938

**Published:** 2015-06-01

**Authors:** Qiangming Liu, Jiancai Qin, Tianwei Li, Erbao Liu, Dejia Fan, Wisdom Mawuli Edzesi, Jianhai Liu, Jianhua Jiang, Xiaoli Liu, Lianjie Xiao, Linglong Liu, Delin Hong

**Affiliations:** 1 State Key Laboratory of Crop Genetics and Germplasm Enhancement, Nanjing Agricultural University, Nanjing, 210095, China; 2 Rice Research Institute, Anhui Academy of Agricultural Sciences, Hefei, 230031, China; Nanjing Forestry University, CHINA

## Abstract

The efficiency of hybrid seed production can be improved by increasing the percentage of exserted stigma, which is closely related to the stigma length in rice. In the chromosome segment substitute line (CSSL) population derived from Nipponbare (recipient) and Kasalath (donor), a single CSSL (SSSL14) was found to show a longer stigma length than that of Nipponbare. The difference in stigma length between Nipponbare and SSSL14 was controlled by one locus (*qSTL3*). Using 7,917 individuals from the SSSL14/Nipponbare F_2_ population, the *qSTL3* locus was delimited to a 19.8-kb region in the middle of the short arm of chromosome 3. Within the 19.8-kb chromosome region, three annotated genes (*LOC_Os03g14850*, *LOC_Os03g14860* and *LOC_Os03g14880*) were found in the rice genome annotation database. According to gene sequence alignments in *LOC_Os03g14850*, a transition of G (Nipponbare) to A (Kasalath) was detected at the 474-bp site in CDS. The transition created a stop codon, leading to a deletion of 28 amino acids in the deduced peptide sequence in Kasalath. A T-DNA insertion mutant (05Z11CN28) of *LOC_Os03g14850 *showed a longer stigma length than that of wild type (Zhonghua 11), validating that *LOC_Os03g14850 *is the gene controlling stigma length. However, the Kasalath allele of *LOC_Os03g14850 *is unique because all of the alleles were the same as that of Nipponbare at the 474-bp site in the CDS of *LOC_Os03g14850 *among the investigated accessions with different stigma lengths. A gene-specific InDel marker LQ30 was developed for improving stigma length during rice hybrid breeding by marker-assisted selection.

## Introduction

Rice (*Oryza sativa* L.) is a main cereal crop for billions of people worldwide. In China, the rice planting area is approximately 3.2×10^7^ ha each year, and hybrid rice is planted in over half of the total rice growing area. Whereas *indica* hybrid rice accounts for 80% of the planting area of *indica* rice, *japonica* hybrid rice accounts for only 3% of the planting area of *japonica* rice [1]. A main limiting factor hindering the extension of the *japonica* hybrid rice area is the low yield of hybrid seed production. A low yield of F_1_ seed production is mainly caused by a low outcrossing rate of the maternal parent (CMS lines or TGMS lines) in the F_1_ seed production field.

Stigma exsertion is a major factor that can increase the opportunity for outcrossing pollination [2, 3, 4]. To date, 38 QTLs affecting stigma exsertion have been identified, and they are distributed on all 12 rice chromosomes [5, 6, 7, 8, 9, 10]. However, only 3 of the 38 QTLs explained more than 10% of the phenotypic variation. Stigma exsertion is easily affected by many environmental conditions (wind, temperature, humidity, physical interruption, etc.) during the flowering period [10]. Many studies have shown that stigma exsertion and stigma length are highly positive correlated [2, 8, 11, 12, 13]. As stigma length is less subject to external conditions, we consider stigma length a more reliable measurement trait than stigma exsertion in studies of mining favorable alleles for improving the outcrossing rate of the maternal parent. To our knowledge, 22 QTLs that condition stigma length have been detected previously; 16 of these QTLs have been shown to explain more than 10% of the phenotypic variation [6, 10, 14].

In the aforementioned studies, nonpermanent segregating populations, such as F_2_ populations, and permanent populations, such as backcross inbred lines (BILs), recombinant inbred lines (RILs) or double haploid lines (DH), were most often used [5, 6, 7, 8, 9, 10, 14]. Unfortunately, when these populations are used, it is difficult to either make repeated observations (for F_2_) or exclude the epistatic effects of various chromosome segments within the same genetic background (for BILs, RILs and DH). These difficulties may explain why no gene cloning/fine mapping of stigma exsertion or stigma length has yet been reported. Chromosome segment substitution lines (CSSLs), in which each line carries a single or a few defined chromosome segments of the donor genome with a pure genetic background from a recurrent genotype, are a powerful tool to conduct QTL mapping with improved mapping precision [15]. Several QTLs have been cloned using CSSL, including *grain size 3* (*GS3*) and *grain wide 5* (*GW5*) for grain size and weight, respectively [16, 17, 18].

**Fig 1:**
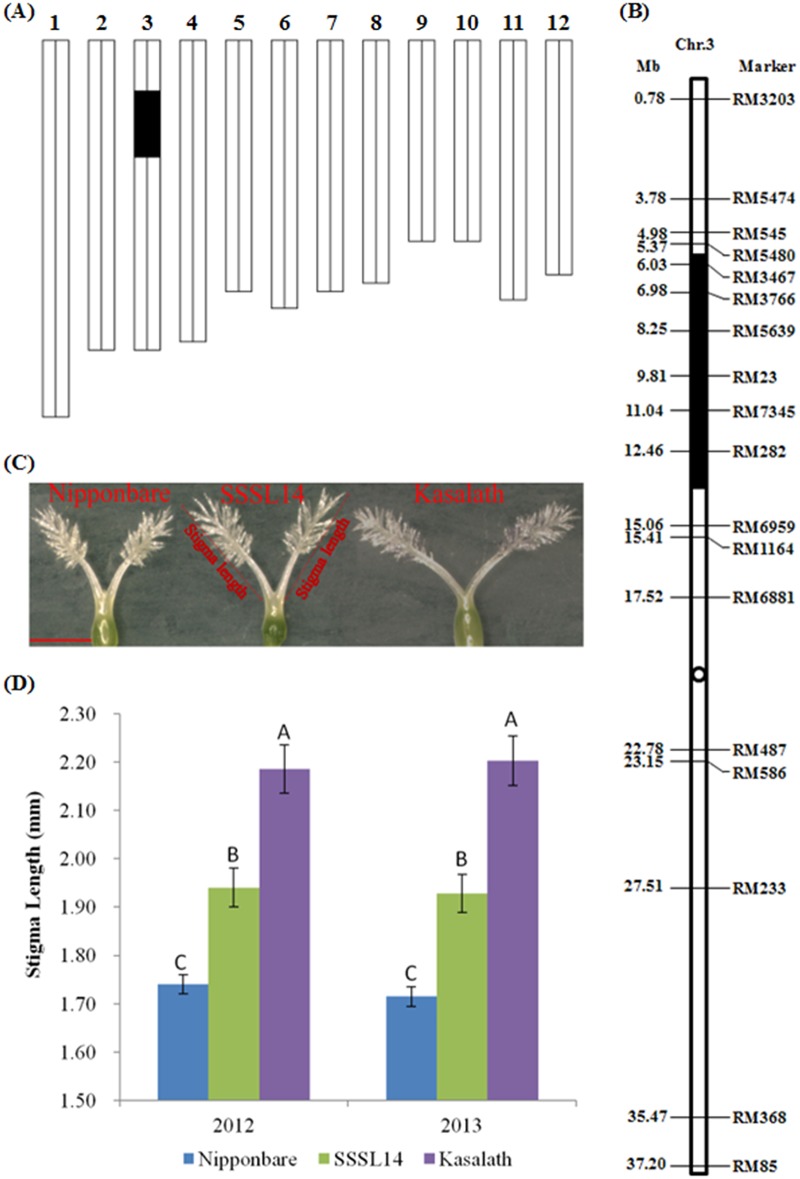
Genotypic and phenotypic performance of the parents. (A) Graphical genotype of SSSL14. The *black bar* indicates the fragment from Kasalath, and the remaining was derived from Nipponbare. (B) Chromosome map based on SSR markers of chromosome 3 of SSSL14. The *black circle* marks the position of the centromere. (C) Stigma morphology of Nipponbare, SSSL14 and Kasalath. *Bar* = 1 mm. The two thin lines aside the stigma of SSSL14 show the method used to measure stigma length. (D) Stigma length of Nipponbare, SSSL14 and Kasalath over two years. A, B and C were ranked by Duncan’s test at *P*<0.01.

In the region of C563-C63 on chromosome 3, an allele from Kasalath was previously identified to increase the percentage of exserted stigma (*qPES-3*) [9] and stigma length (*qSTL3*) [14] using the BIL population derived from Nipponbare/Kasalath//Nipponbare (S1 Table). Accordingly, the C563-C63 region might be a useful fragment to improve the stigma traits of the maternal parent in *japonica* hybrid rice. A single segment substitution line (named SSSL14) containing only one fragment from the donor parent Kasalath in the C563-C63 region was obtained from a set of CSSLs using Nipponbare as the recipient (Fig 1A). In this paper, we finely mapped *qSTL3* using an F_2_ population derived from SSSL14/Nipponbare. Next, we further analyzed candidate genes of *qSTL3* through gene sequence alignments, real time quantitative RT-PCR and T-DNA insertion mutant analysis with the aim of providing a genetic basis for cloning the gene. Additionally, a gene-specific marker was developed for improving the stigma length of the maternal parent, thereby increasing the outcrossing rate of the maternal parent in a *japonica* hybrid seed production field.

## Materials and Methods

### Plant materials and cultivation

Nipponbare, Kasalath, SSSL14, the F_1_ plants of SSSL14/Nipponbare and the F_2_ population derived from SSSL14/Nipponbare were used to finely map *qSTL3*. The seeds of Nipponbare, Kasalath and SSSL14 were provided by the Rice Genome Resource Center (RGRC, http://www.rgrc.dna.affrc.go.jp/stock.html), Japan. The recipient parent, *japonica* cultivar Nipponbare, has a short stigma, while the donor parent, *indicia* cultivar Kasalath, has a long stigma. SSSL14 was a single segment substitution line with only one introgressed segment in the middle of the short arm of chromosome 3 (Fig 1A, 1B and S1 Fig). Compared to Nipponbare, SSSL14 showed a longer stigma, higher stigma exsertion, more slender grain, higher plant height and later heading date (Fig 1C, 1D and S2 Fig). The F_1_ hybrid seeds between SSSL14 and Nipponbare were harvested in October of 2011. The F_2_ seeds set on F_1_ plants were harvested in October of 2012. A secondary segregating population composed of approximately 8,000 F_2_ plants was cultivated in the rice growing season of 2013.

Seeds of a T-DNA insertion mutant (05Z11CN28) and its wild type (Zhonghua 11) were bought from the National Center of Plant Gene Research (NCPGR) at Huazhong Agricultural University, Wuhan City, Hubei Province, China in 2014.

A population composed of 80 rice accessions collected from different regions of China, Vietnam and Japan were employed to investigate the allele variations of the target gene locus.

All of the plant materials were planted in a paddy field at the experimental farm of Nanjing Agricultural University, Nanjing City, Jiangsu Province, China (118.64°E, 32.07°N), with a density of 17 cm × 25 cm. Except for the F_2_ population, all of the materials were planted in a randomized complete block design with two replications. Forty plants (five rows, with eight plants per row) per replication were cultivated for each material. Crop management followed commercial rice production practices.

### Phenotypic evaluation

For all of the planted materials, the stigma length was evaluated. In the F_2_ population of SSSL14/Nipponbare, eight flowering glumes were collected at the full-bloom stage from the highest panicle on each individual and fixed in acetic alcohol (ratio of acetic acid to ethanol = 1:3, v/v). Next, the pistil of each glume was observed under a stereomicroscope (Guangzhou Micro-shot Technology Co., Ltd., Guangzhou, Guangdong Province, China), and the stigma length was measured with a micrometer (Fig 1C). Next, the mean stigma length of the plant was calculated from the measurements of 16 stigmas (2 stigmas per pistil). Finally, all F_2_ individuals were grouped into three phenotypes according to stigma length (<1.73 mm for the Nipponbare homozygote, 1.74–1.88 mm for the heterozygote, and >1.89 mm for the SSSL14 homozygote). For the other materials, three plants were evaluated in each line in each replication.

Stigma exsertion of Nipponbare, SSSL14 and Kasalath was investigated 10 days after heading. Stigmas that remained outside of the glume after the palea and lemma closed were defined as exserted stigmas. The percentage of exserted stigmas was calculated by the method proposed by Miyata et al [8] with minor modifications. In brief, the percentage of exserted stigmas was defined as the rate (%) of exserted stigmas over the total stigmas on a entire panicle. In addition, to characterizing Nipponbare, the F_1_ of SSSL14/Nipponbare, SSSL14, Kasalath, Zhonghua 11 (wild type) and the mutant line, seven other traits, i.e., grain length, grain width, grain thickness, thousand-grain weight, plant height, days to heading and tiller number per plant were evaluated using 10 plants each.

### DNA extraction and molecular marker genotyping

Microquantities of DNA were extracted from fresh leaves of individuals from the F_2_ population using the method reported by Monna et al [19]. PCR was performed in a 10-μL reaction volume containing 1.5 μL of 20.0 ng/μL template DNA, 1.0 μL of 10× PCR buffer, 0.25 μL of 1.0 pmol/μL dNTPs, 1.5 μL of 2.0 pmol/μL primer pairs, 0.06 μL of 5.0 U/μL *Taq* DNA polymerase and 5.69 μL of ddH_2_O. The amplification regime comprised an initial denaturation step (94°C for 5 min), followed by 32 cycles of 94°C for 30 s, 55°C for 30 s, and 72°C for 1 min. The reactions were completed with a final extension step of 72°C for 7 min. The PCR products were separated by electrophoresis through 8% non-denaturing polyacrylamide gels at a voltage of 180 V for approximately 100 min and then visualized by silver staining [20].

### Conversion of RFLP markers to SSR markers on chromosome 3

Because the only RFLP marker information for SSSL14 was available in RGRC (S1 Fig), SSR markers were used to genotype chromosome 3 of SSSL14. A total of 21 of the 67 selected SSR primers on chromosome 3 revealed polymorphisms between Nipponbare and Kasalath, and these primers were used to genotype and construct the chromosome map based on SSR markers of chromosome 3 of SSSL14 (Fig 1B). The donor fragment was positioned by the SSR markers RM3467 and RM282 at an average length of 8.06 Mb.

### Linkage analysis of *qSTL3*

A group of 220 plants (POP1) were randomly selected from the F_2_ population and used to construct a molecular linkage map with 6 polymorphic SSR markers and an average distribution in the introgressed fragment region for primary QTL analysis. The linkage map was constructed using MapMaker3.0/EXP version 3.0 [21]. QTL analysis of *qSTL3* was performed by the inclusive composite interval mapping method in IciMapping version 3.3 (www.isbreeding.net/) based on a stepwise linear regression model [22].

### Fine mapping of *qSTL3*

Additional SSR markers in the preliminary mapping region were selected from previously published SSR primers [23], and seven InDel markers (Table 1) were developed by sequence alignment of Nipponbare and Kasalath sequences, which were available from the Rice Annotation Project Database (RAP-DB, http://rapdb.dna.affrc.go.jp/). These informative markers were anchored on bacterial artificial chromosome (BAC) or P1-derived artificial chromosome (PAC) clones of the reference Nipponbare genome (http://agri-trait.dna.affrc.go.jp/). Next, the genotypes of these markers in individual plants were determined, and the recombinants were identified.

**Table 1:** Primer sequences designed in this study.

Marker	Marker Type/Gene ID	Purpose	Forward primer (5’-3’)	Reverse primer (5’-3’)
**LQ12**	InDel	Fine mapping	TCGTACATCAATCAAACATGC	GTGCGCCACCTTTATTTTA
**LQ14**	InDel	Fine mapping	TAACATCCGGTCAAACATCC	GCTCAACAGTCAACATCTTC
**LQ17**	InDel	Fine mapping	GTAATGAGGTGACCGAACC	AGATCAGAAATCCCAGTGC
**LQ21**	InDel	Fine mapping	GGAAACACGGCTAAAGTTTG	TTATTGGTTCGACCAGCCAT
**LQ23**	InDel	Fine mapping	AGGTGCGTTTAGTTAGTAGC	AGTTTTGATGTGATGAAAAAGTT
**LQ26**	InDel	Fine mapping	CTGGAAAAGCGAAATCCAATA	TTTTGTGTCTTTTACGGTGT
**LQ29**	InDel	Fine mapping	ATACATCTCTAGCTGCTCCA	CAGCTGAGCCATCATATAGT
**RT2**	*LOC_Os03g14850*	Real time RT-PCR	GCGCCACACTACCATCTTCA	CCCGCTTTGGGTTGAGCTA
**RT5**	*LOC_Os03g14860*	Real time RT-PCR	GCGGTGGTTACACAGCGATA	TTCTCAACAAGGTGCCCACA
**RT20**	*LOC_Os03g14880*	Real time RT-PCR	TCACCAGTTTGAGCCGAAGT	TCATTGTCGCCACCCTTCAA
**RT23**	*18S rRNA*	Real time RT-PCR	ATGATAACTCGACGGATCGC	CTTGGATGTGGTAGCCGTTT
**LQ30**	InDel	MAS	TTGCCAAATGATGAGAACAAA	GTCTAAAGAGAACTGAGCACT

### Sequence alignment of candidate genes

Based on the fine mapping result, the annotated genes of *qSTL3* were searched against RAP-DB. Subsequently, sequences of the candidate genes of the original parents, Nipponbare and Kasalath, were further analyzed by DNAMAN software (version 7.0.2.176, Lynnon Biosoft, Quebec, Canada).

### Expression analysis of candidate genes

According to landmark developmental events, the development of the stigma (an important part of the pistil) starts at *Stage In7* (differentiation of floral organs) and ends at *Stage In8* (rapid elongation of rachis and maturation of reproductive organs) [24]. Therefore, frozen young panicles (*Stage In7* to *Stage In8*, with a panical length of 5 mm to 100 mm) of Nipponbare and SSSL14 during the pre-heading stage were employed to isolate total RNA using the Ultrapure RNA Kit (CoWin Biotech Co., China). RNase-free DNase I treatment was performed to remove any genomic DNA contamination. First-strand cDNA was reverse transcribed from 1 μg of RNA using a HiFiScript Quick gDNA Removal cDNA Kit (CoWin Biotech Co., China). The *18S rRNA* was used as an internal control to normalize gene expression. Real time quantitative RT-PCR was performed in a 96-well thermocycler (Roche Applied Science LightCycler 480) using the AceQ qPCR Kit (Vazyme). The cycling conditions were 5 min at 95°C followed by 40 cycles of amplification (95°C for 10 s and 60°C for 30 s). The primers are listed in Table 1. Relative gene expression of the target gene was calculated using the following equation: *Exp* = 2^-Δ*Ct*^, where Δ*Ct* = *Ct*_*target gene*_-*Ct*_18*S rRNA*_.

### Analysis of T-DNA insertion mutants

The insertion mutant lines of the candidate genes were searched against the Rice Functional Genomic Express Database (RiceGE, http://signal.salk.edu/cgi-bin/RiceGE). The mutant lines were collected from the Rice Mutant Database (RMD) [25]. For mutant genotyping, gene-specific PCR primers (P1: GTCAGTCAGCCCCAATCCAA and P2: GCCATGCGTGTCCATGTTTT) flanking the T-DNA insertion site and a vector border primer (P3: AATCCAGATCCCCCGAATTA) were designed. The expression changes of the genes containing the insertion site were also evaluated by real time quantitative RT-PCR using the total RNA isolated from frozen young panicles (5 mm to 100 mm) of the wild type and mutant during the pre-heading stage.

### Sequencing analysis of the allele variation of *LOC_Os03g14850*

Sequencing analysis of the allele variation of *LOC_Os03g14850* was performed using 80 accessions with various stigma length. Gene-specific PCR primers (P4: TTTGTAGCTCTTCTTCGTTC and P5: GATCAAGCCCAATGCCAACA) were designed to amplify the target DNA fragment. The PCR product was gel-purified and sequenced by GenScript Corporation Ltd., Nanjing, China. Multiple sequence alignment was performed with DNAMAN.

## Results

### Stigma length of SSSL14, Nipponbare, and the F_1_ and F_2_ population of SSSL14/Nipponbare

The stigma length of SSSL14 was significantly longer than that of Nipponbare (i.e., 1.93±0.04 vs. 1.71±0.02 mm) in 2013, which was consistent with the measurements collected in 2012 (Fig 1D). In contrast, the SSSL14/Nipponbare F_1_ exhibited a stigma length intermediate between the two parents (1.80±0.02 mm) in 2013, indicating that the allele for long stigma length exhibits a semi-dominant pattern of expression. Among the random selection of 220 plants in POP1, the phenotypic separation ratio was fitted to 1:2:1 (45 Nipponbare homozygote type: 121 heterozygote type: 54 SSSL14-homozygote type,$\chi^{2}=2.94<\chi_{0.05,\;2}^{2}=5.99$), suggesting that stigma length is controlled by a single gene locus.

### Preliminary mapping of *qSTL3*

**Fig 2:**
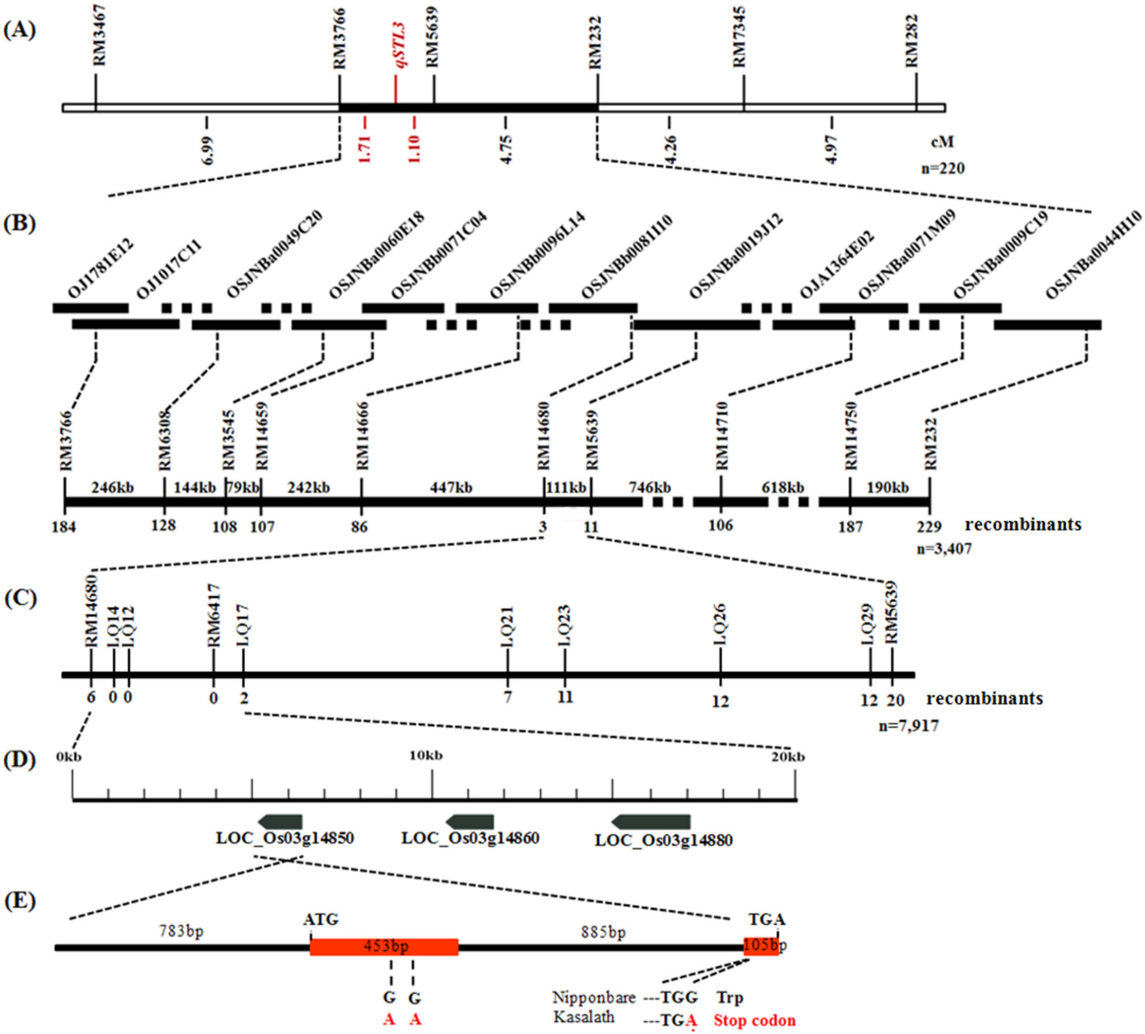
Fine mapming and candidate gene analysis of *qSTL3*. (A) Primary mapping of *qSTL3*. The *qSTL3* was mapped between the SSR markers RM3766 and RM5639 based on 220 plants randomly selected from the SSSL14/Nipponbare F_2_ population. (B) Physical mapping of *qSTL3*. The *qSTL3* was positioned between the SSR markers RM14680 and RM5639 based on 3,407 plants from the SSSL14/Nipponbare F_2_ population. (C) High-resolution mapping of *qSTL3*. The *qSTL3* was narrowed down to a 19.8-kb region between the markers RM14680 and LQ17 on the BAC clones OSJNBb0081I10 and OSJNBa0019J12 using a total of 7,917 plants from the SSSL14/Nipponbare F_2_ population. (D) Candidate region of the *qSTL3* locus and the annotated genes in the Rice Annotation Project Database (RAP-DB, http://rapdb.dna.affrc.go.jp/). (E) The structure of the *LOC_Os03g14850* gene and variation sites. The *orange bar* indicates the exon.

Using the SSR molecular linkage map of chromosome 3 and the stigma length data collected from the 220 individuals in POP1, *qSTL3* was delimited to a 2.81-cM region between RM3766 and RM5639 with an LOD peak of 18.19 (Fig 2A and S3 Fig). This locus explained 33.1% of the phenotypic variance. The allele from SSSL14 increased stigma length.

### Fine mapping of *qSTL3*

A total of 3,407 F_2_ plants, including the 220 POP1 plants, were subjected to marker analysis by scanning RM3766 and RM232 (confidence interval markers of *qSTL3* in preliminary mapping). An analysis of RM3766 identified 184 recombination events between the marker and *qSTL3* on one side, while an analysis of RM232 detected 229 recombination events between the marker and *qSTL3* on the other side. The SSR markers RM6308, RM3545, RM14659, RM14666 and RM14680 revealed 128, 108, 107, 86 and 3 recombinants, respectively, while RM14750, RM14710 and RM5639 showed 187, 106 and 11 recombinants, respectively, on the other side (Fig 2B). Therefore, *qSTL3* was mapped to a 110-kb DNA region between RM14680 and RM5639.

The SSR markers RM14680 and RM5639 were further used to identify recombination break points, and 26 individuals were screened from 7,917 F_2_ plants, including the former 3,407 plants. Seven newly developed InDel markers and one SSR marker were used to narrow the region of *qSTL3*. LQ14, LQ12 and RM6417 were found to co-segregate with *qSTL3*. Six recombinants were detected between RM14680 and *qSTL3*, while 20, 12, 12, 11, seven and two recombinants were revealed at RM5639, LQ29, LQ26, LQ23, LQ21 and LQ17, respectively, on the other side (Fig 2C). Finally, *qSTL3* was localized to a 19.8-kb interval defined by RM14680 and LQ17 on the BAC clones OSJNBb0081I10 and OSJNBa0019J12.

### Sequence alignment of candidate genes

According to RAP-DB, there were three annotated genes (*LOC_Os03g14850*, *LOC_Os03g14860* and *LOC_Os03g14880*) in the 19.8-kb region (Fig 2D). *LOC_Os03g14850* was identified as a MADS-box family gene with a M-alpha type-box, *LOC_Os03g14860* encodes a protein that contains a G-patch domain, and *LOC_Os03g14880* expresses a hypothetical protein.

To recognize the differences in candidate genes between Nipponbare and SSSL14, pairwise sequence alignments of these genes were performed. For *LOC_Os03g14850*, 11 variants, including one three-base deletion and 10 single-base substitutions, were identified in the genic sequence of Nipponbare vs. Kasalath (S4A Fig). Among 10 single-base substitutions, three were in the coding sequence (CDS). One of these variations involved a G (Nipponbare) to A (Kasalath) change at the 474-bp site in the CDS that created a stop codon, leading to premature termination at codon 158 (Fig 2E and S4B Fig) and a deletion of 28 amino acids (AA) in the deduced peptide sequence (S4C Fig). For *LOC_Os03g14860*, Kasalath possessed four single-base substitutions compared with Nipponbare (S4D and S4E Fig), leading to 3 AA changes between Nipponbare and Kasalath (S4F Fig). For *LOC_Os03g14880*, one three-base insertion, one single-base deletion, and two single-base transitions were detected between Kasalath and Nipponbare (S4G Fig), but no variation was found in the CDS and AA sequences (S4H and S4I Fig).

### Expression differences of candidate genes in parents

**Fig 3:**
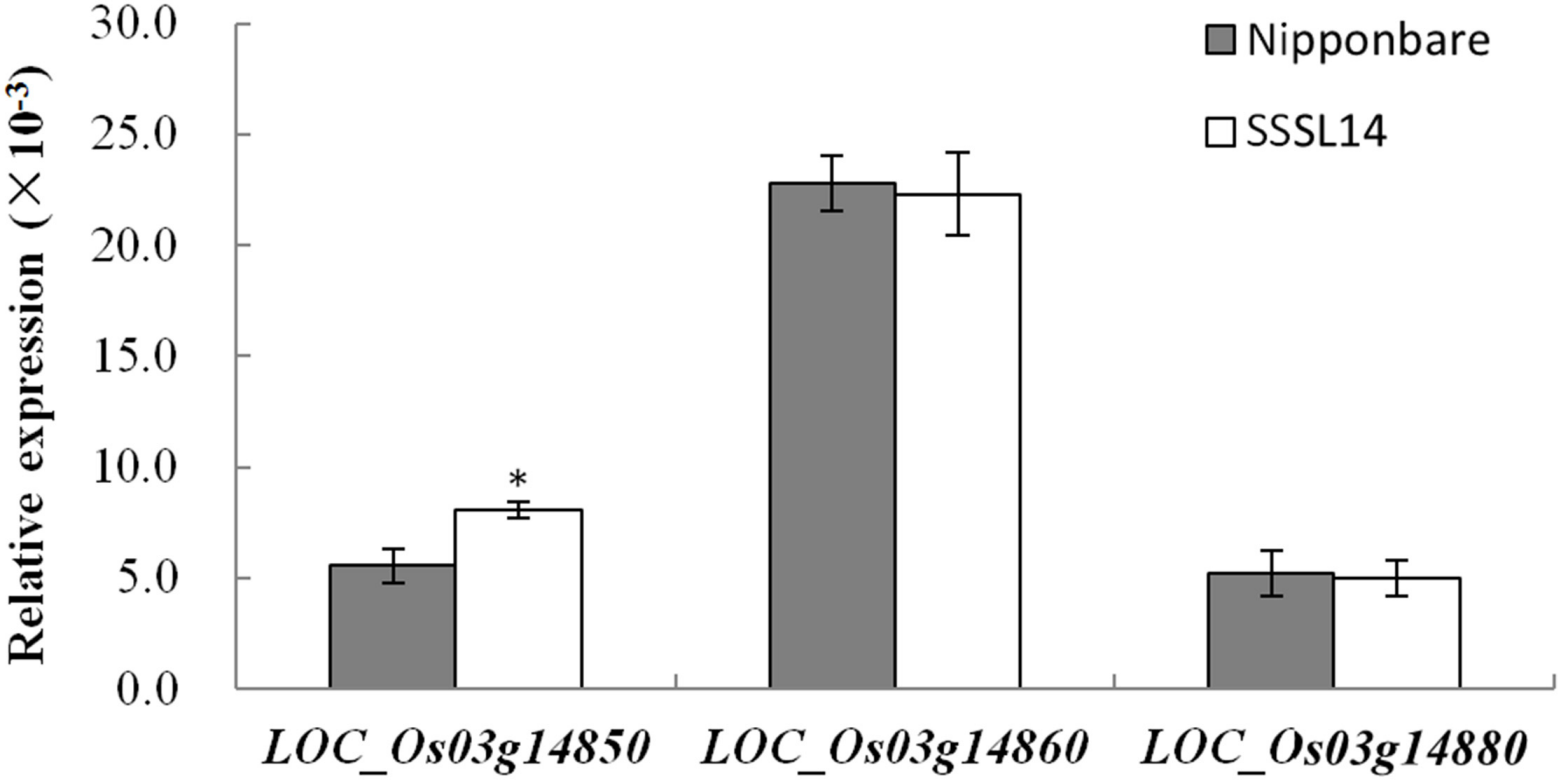
Expression levels of candidate genes by real time quantitative RT-PCR. RNA was isolated from frozen young panicles (5 mm to 100 mm) in Nipponbare and SSSL14. * indicates significant difference between parents at α = 0.05 probability level. Values are the mean ± SD with three biological replicates.

To further analyze the candidate genes, real time quantitative RT-PCR was employed to detect the expression differences between the parents. The results showed that, the expression level of *LOC_Os03g14850* in SSSL14 was significantly up-regulated (1.46-fold) compared to that of Nipponbare, while no differential expression of *LOC_Os03g14860* and *LOC_Os03g14880* was observed in Nipponbare and SSSL14 (Fig 3).

### Analysis of T-DNA insertion mutant of *LOC_Os03g14850*

**Fig 4:**
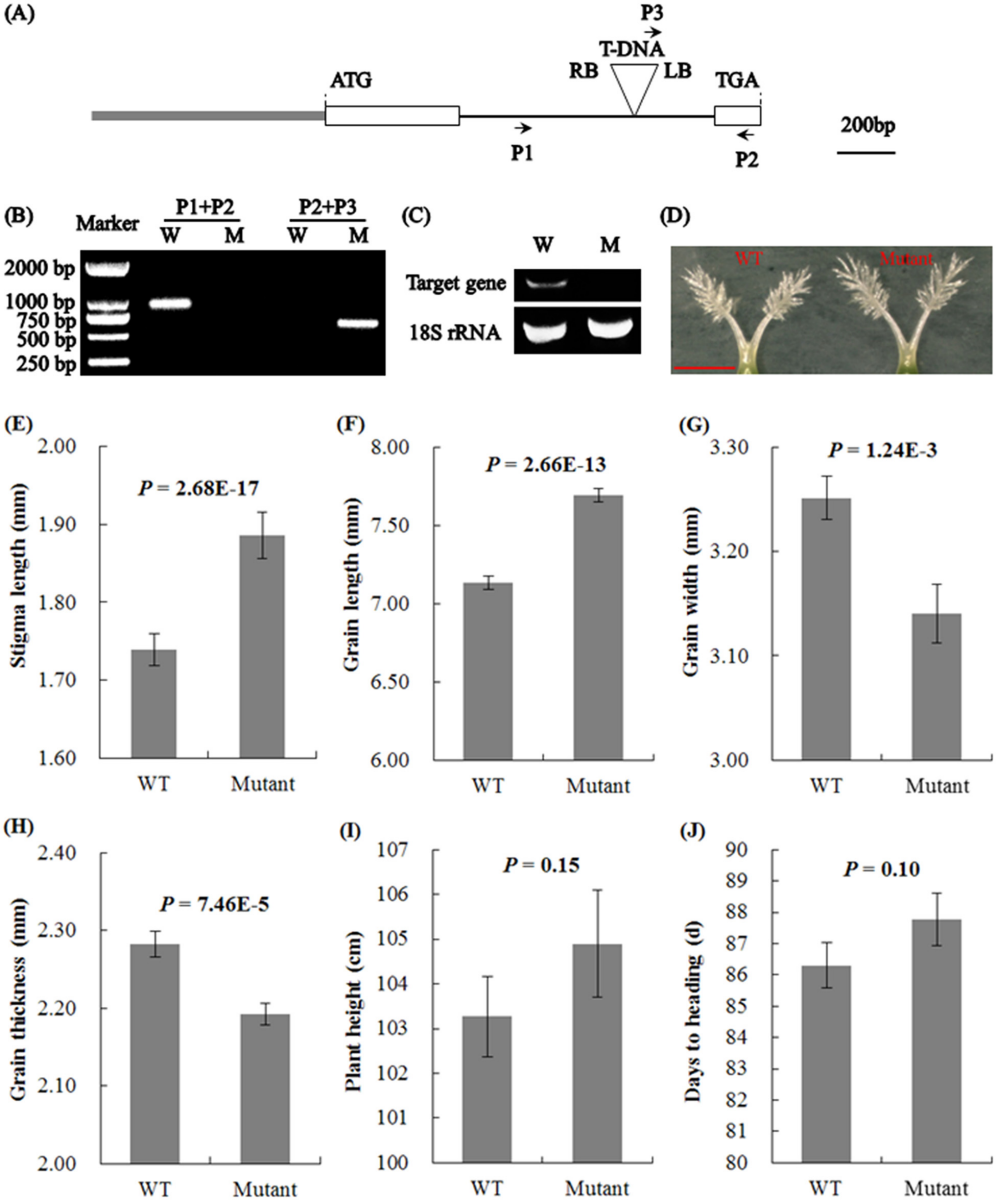
T-DNA insertion mutant analysis of *LOC_Os03g14850*. (A) *LOC_Os03g14850* gene structure and T-DNA insertion site. White boxes, thin lines and gray boxes represent exons, introns and UTRs, respectively. LB and RB represent the left and right border of the T-DNA, respectively. P1 and P2 represent the gene-specific PCR primers flanking the T-DNA insertion site and P3 represents the vector border primer. (B) PCR genotyping results. W and M indicate the wild type and homozygous mutant, respectively. (C) Real time quantitative RT-PCR result of *LOC_Os03g14850* in wild type and the homozygous mutant. The target gene was *LOC_Os03g14850*. The *18S rRNA* gene was the control. (D) Stigma phenotype of wild type and mutant. *Bar* = 1 mm. (E-I) Comparison of the stigma length, grain length, grain width, grain thickness, plant height and days to heading of wild type and mutant. A *t* test was performed between wild type and mutant. All data are given as the mean ± SD.

For studying the biological function of the three candidate genes, mutants were searched against the RiceGE. As a result, only one putative T-DNA mutant line (05Z11CN28) was found, and the insertion site was in the intron of *LOC_Os03g14850* (Fig 4A). Through two sets of PCR for genotyping, the insertion site of this mutant line was confirmed (Fig 4B). The real time quantitative RT-PCR results showed that the *LOC_Os03g14850* transcript was absent in the homozygous mutant (Fig 4C). The stigma length of the homozygous mutant (1.89±0.03 mm) was increased by 8.62% compared with that of the wild type (1.74±0.02 mm) (Fig 4D and 4E). The sequence analysis of *LOC_Os03g14850* of Zhonghua 11 showed no gene sequence difference between Nipponbare and Zhonghua 11 (S5 Fig). The results indicated that *LOC_Os03g14850* was the gene controlling stigma length.

Compared to wild type, the grain length of the mutant was 7.8% longer (Fig 4F), whereas the grain width and grain thickness showed decreases of approximately 3.4% and 3.9%, respectively (Fig 4G and 4H). For plant height and days to heading, no significant differences were observed between wild type and mutant (Fig 4I and 4J). This result suggested that the gene locus *LOC_Os03g14850* also had pleiotropic effects on grain size traits.

### Analysis of allele variation of *LOC_Os03g14850*

**Fig 5:**
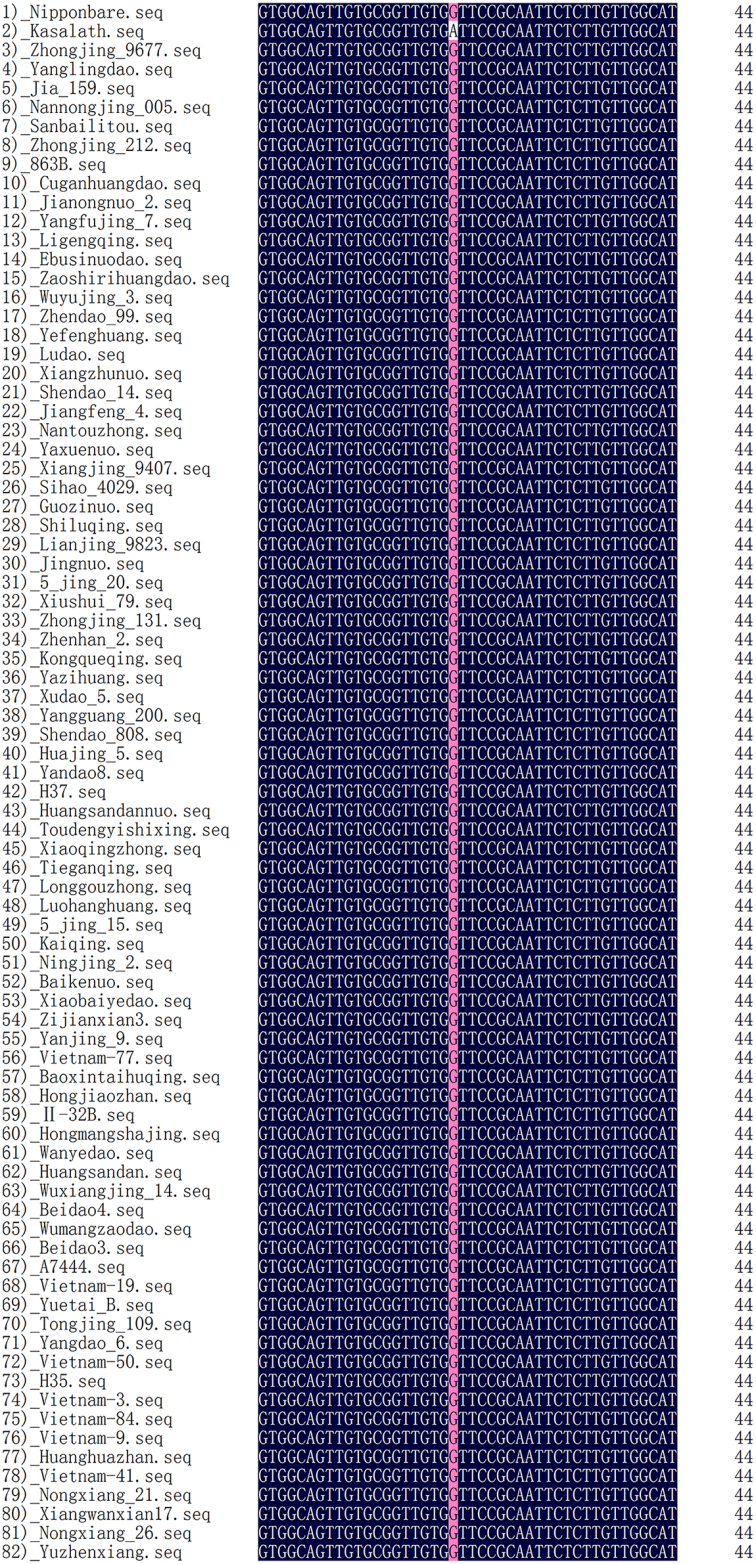
Sequence alignment of Nipponbare, Kasalath and the 80 accessions with different stigma lengths No.1-2 are the original parents of our fine mapping population, No.3-42 are the short stigma accessions, and No.43-82 are the long stigma accessions.

The sequencing analysis showed that all alleles were the same as that of Nipponbare at the 474-bp site in the CDS of *LOC_Os03g14850* among the 80 accessions with various stigma lengths (Fig 5, S2 Table). This result indicated that the transition of G (Nipponbare) to A (Kasalath) created a unique allele of *LOC_Os03g14850* in Kasalath.

### Development of a gene-specific molecular marker

An InDel marker LQ30 was developed according to the three-base deletion in the intron of *LOC_Os03g14850* in Kasalath comparing to Nipponbare (Table 1). The profile amplified by LQ30 displayed that all the marker genotypes of the 80 accessions were the same as that of Nipponbare (S6 Fig). This result is consistent with that of the sequencing. Therefore, the newly developed InDel marker LQ30 is a gene-specific molecular marker for *LOC_Os03g14850*.

## Discussion

In the present study, we delimited the chromosome segment containing *qSTL3* to a 19.8-kb region for the first time. In our previous study, a QTL that corresponded to the percentage of exserted stigma was detected in the region of C563-C63 on chromosome 3 [9]. In the same chromosome region, there is a gene locus affecting stigma length [14]. A gene locus that mainly regulates grain size, *GS3*, was reported to participate in stigma length and the percentage of exserted stigma [26, 27]. However, the physical position (17.36 Mb from the end of the short arm of chromosome 3) of *GS3* is far from that of the C563-C63 region (6.78 Mb-8.46 Mb from the end of the short arm of chromosome 3) (S1 Fig). We predicted there is a gene cluster controlling stigma length on the short arm of chromosome 3.

The *LOC_Os03g14850* was validated to be the gene locus controlling the stigma length. Within the fine mapping region, we found three annotated genes, i.e., *LOC_Os03g14850*, *LOC_Os03g14860* and *LOC_Os03g14880*. The *LOC_Os03g14880*, which encodes a putative conserved hypothetical protein with an unknown function, showed no sequence difference in CDS between Nipponbare and Kasalath. The *LOC_Os03g14860*, which is annotated as a G-patch domain-containing protein, was reported to be mainly associated with disease resistance and root development [28, 29, 30]. Furthermore, on the basis of the real time quantitative RT-PCR, no differential expression of *LOC_Os03g14860* and *LOC_Os03g14880* was observed between Nipponbare and SSSL14. The *LOC_Os03g14850* expresses a type I MADS-box protein (OsMADS72) that belongs to a sequence-specific DNA binding transcription factor family and is well known to participate in flower development [31]. Recent studies have suggested that type I MADS-box genes are also important for plant reproduction and development, especially for determining female gametophyte, embryo, and endosperm development in *Arabidopsis* [32, 33, 34, 35]. Sequence alignment of *LOC_Os03g14850* between Nipponbare and Kasalath displayed a nonsyn-SNP at the 474-bp site in the CDS in Kasalath, which results in the deletion of 28 amino acids (AA) in the deduced peptide sequence. Furthermore, the analysis of a T-DNA insertion mutant showed that the gene knockout of *LOC_Os03g14850* leads to a longer stigma. The data demonstrated that *LOC_Os03g14850*, rather than *LOC_Os03g14860* and *LOC_Os03g14880*, was the gene controlling stigma length.

The Kasalath allele of *LOC_Os03g14850* was a unique allele, as no allele variation was found at this gene locus among the investigated accessions with different stigma length. Kasalath belongs to the *indica* sub-species of the *aus* group, which were mainly grown under rainfed conditions in Indian and Bangladesh [36]. As a result of specific growing environment, the *aus* group rices have high genome diversity [36, 37]. Hence, more accessions with the specific Kasalath-type allele of *LOC_Os03g14850* are to be expected in *aus* group rice.

The newly developed gene-specific InDel marker LQ30 of *LOC_Os03g14850* will be useful in breeding program for improving the stigma length of the maternal parent by MAS. In addition, *LOC_Os03g14850* showed pleiotropic effects on grain size traits (Fig 4F–4J). Intriguingly, the cloned gene *GS3* also showed pleiotropic effects on grain size and stigma length [27]. We predict that breeding a long stigma *japonica* maternal parent may result in a slender grain shape.

## Conclusions

The unique Kasalath allele of *LOC_Os03g14850* on chromosome 3 may increase stigma length in rice. The newly developed gene-specific InDel marker LQ30 could be used to improve the stigma length of the maternal parent by MAS.

## Supporting Information

S1 Fig RFLP graphical genotype of chromosome 3 of SSSL14. The black bar indicates the fragment from Kasalath, and the remaining was derived from Nipponbare. The black circle marks the position of the centromere.
(PDF)

S2 Fig Phenotypic evaluations of eight agronomic traits for Nipponbare and SSSL14. Comparison of the percentage of exserted stigma (A), grain length (B), grain width (C), grain thickness (D), thousand-grain weight (E), plant height (F), days to heading (G) and tiller number (H) of Nipponbare and SSSL14. All data are given as the mean ± SD (summer 2013, Nanjing, Jiangsu, China). The *P* value for each trait was obtained from a *t* test between SSSL14 and Nipponbare.
(PDF)

S3 Fig QTL mapping of *qSTL3* based on 220 plants randomly selected from the SSSL14/Nipponbare F_2_ population. (PDF)

S4 Fig Sequence alignments of three candidate genes in Nipponbare and Kasalath. (A)-(C) Gene sequence, coding sequence and amino acid sequence of *LOC_Os03g14850*, respectively. (D)-(F) Gene sequence, coding sequence and amino acid sequence of *LOC_Os03g14860*, respectively. (G)-(I) Gene sequence, coding sequence and amino acid sequence of *LOC_Os03g14880*, respectively.
(PDF)

S5 Fig Sequence alignment of *LOC_Os03g14850* in Nipponbare and Zhonghua 11. (PDF)

S6 Fig The profile amplified by the gene-specific marker LQ30 using total DNA of Nipponbare, Kasalath and the 80 accessions with different stigma lengths. M indicates the DNA marker ladder, N and K indicate Nipponbare and Kasalath, No.3-42 indicate the short stigma accessions, and No.43-82 indicate the long stigma accessions.
(PDF)

S1 Table QTL information on the percentage of exserted stigma and stigma length identified on the short arm of chromosome 3. ^1^ Both *qPES-3* and *qSTL*3(t) were identified in the BIL population derived from Nipponbare/Kasalath//Nipponbare. ^2^ All QTLs detected in this reference were not named. We named the QTL for stigma length as *qSTL3*(t).
(PDF)

S2 Table Basic information of the accessions used for sequencing analysis. No.1-2 are the original parents of the fine mapping population, No.3-42 are the accessions with short stigma length, and No.43-82 are the accessions with long stigma length. All stigma length data are given as the mean ± SD (summer 2014, Nanjing, Jiangsu, China). *, ** indicate the least significant difference at a 0.05 and 0.01 probability level compared with Nipponbare by *t* test, respectively.
(PDF)
