# Supplementary material for: Fine Mapping and Candidate Gene Analysis of qSTL3, a Stigma Length-Conditioning Locus in Rice (Oryza sativa L.)
Source: PLoS One. 2015 Jun 1;10(6):e0127938. doi: 10.1371/journal.pone.0127938 (PMC4452489; doi:10.1371/journal.pone.0127938)
Supplement: S1 Fig — The black bar indicates the fragment from Kasalath, and the remaining was derived from Nipponbare. The black circle marks the position of the centromere. (PDF) [file pone.0127938.s001.pdf]

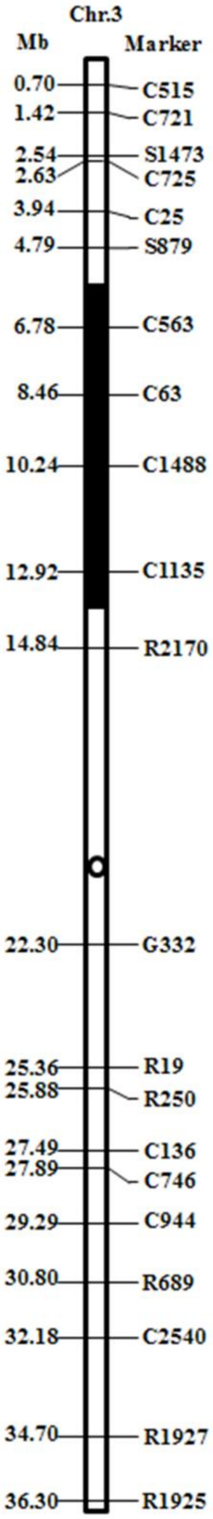

S1 Fig. Chromosome map based on RFLP markers of chromosome 3 of SSSL14.

The black bar indicates the fragment from Kasalath, and the remaining was derived from Nipponbare. The black circle marks the position of the centromere.
