# Supplementary material for: Fine Mapping and Candidate Gene Analysis of qSTL3, a Stigma Length-Conditioning Locus in Rice (Oryza sativa L.)
Source: PLoS One. 2015 Jun 1;10(6):e0127938. doi: 10.1371/journal.pone.0127938 (PMC4452489; doi:10.1371/journal.pone.0127938)
Supplement: S2 Fig — Comparison of the percentage of exserted stigma (A), grain length (B), grain width (C), grain thickness (D), thousand-grain weight (E), plant height (F), days to heading (G) and tiller number (H) of Nipponbare and SSSL14. All data are given as the mean ± SD (summer 2013, Nanjing, Jiangsu, China). The P value for each trait was obtained from a t test between SSSL14 and Nipponbare. (PDF) [file pone.0127938.s002.pdf]

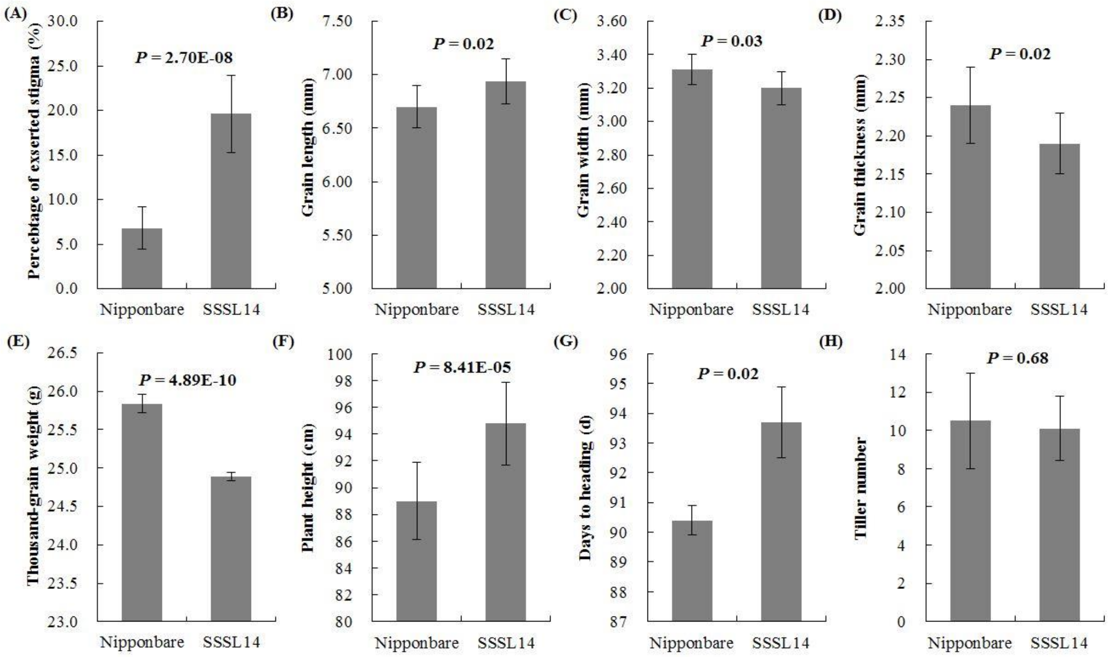

S2 Fig. Phenotypic evaluations of eight agronomic traits for Nipponbare and SSSL14. Comparison of the percentage of exserted stigma (A), grain length (B), grain width (C), grain thickness (D), thousand-grain weight (E), plant height (F), days to heading (G) and tiller number (H) of Nipponbare and SSSL14. All data are given as the mean ± SE (summer 2013, Nanjing, Jiangsu, China). Each *P* value for each trait was obtained from a *t* test between Nipponbare and SSSL14.
