# Supplementary material for: Fine Mapping and Candidate Gene Analysis of qSTL3, a Stigma Length-Conditioning Locus in Rice (Oryza sativa L.)
Source: PLoS One. 2015 Jun 1;10(6):e0127938. doi: 10.1371/journal.pone.0127938 (PMC4452489; doi:10.1371/journal.pone.0127938)
Supplement: S3 Fig — (PDF) [file pone.0127938.s003.pdf]

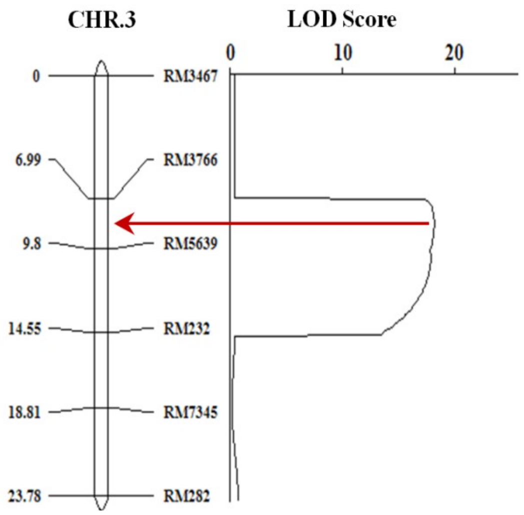


**S3 Fig. QTL mapping of *qSTL3* based on the 220 plants randomly selected from SSSL14/Nipponbare $F_2$ population.**
