# Supplementary material for: Fine Mapping and Candidate Gene Analysis of qSTL3, a Stigma Length-Conditioning Locus in Rice (Oryza sativa L.)
Source: PLoS One. 2015 Jun 1;10(6):e0127938. doi: 10.1371/journal.pone.0127938 (PMC4452489; doi:10.1371/journal.pone.0127938)
Supplement: S4 Fig — (A)-(C) Gene sequence, coding sequence and amino acid sequence of LOC_Os03g14850, respectively. (D)-(F) Gene sequence, coding sequence and amino acid sequence of LOC_Os03g14860, respectively. (G)-(I) Gene sequence, coding sequence and amino acid sequence of LOC_Os03g14880, respectively. (PDF) [file pone.0127938.s004.pdf]

(A)

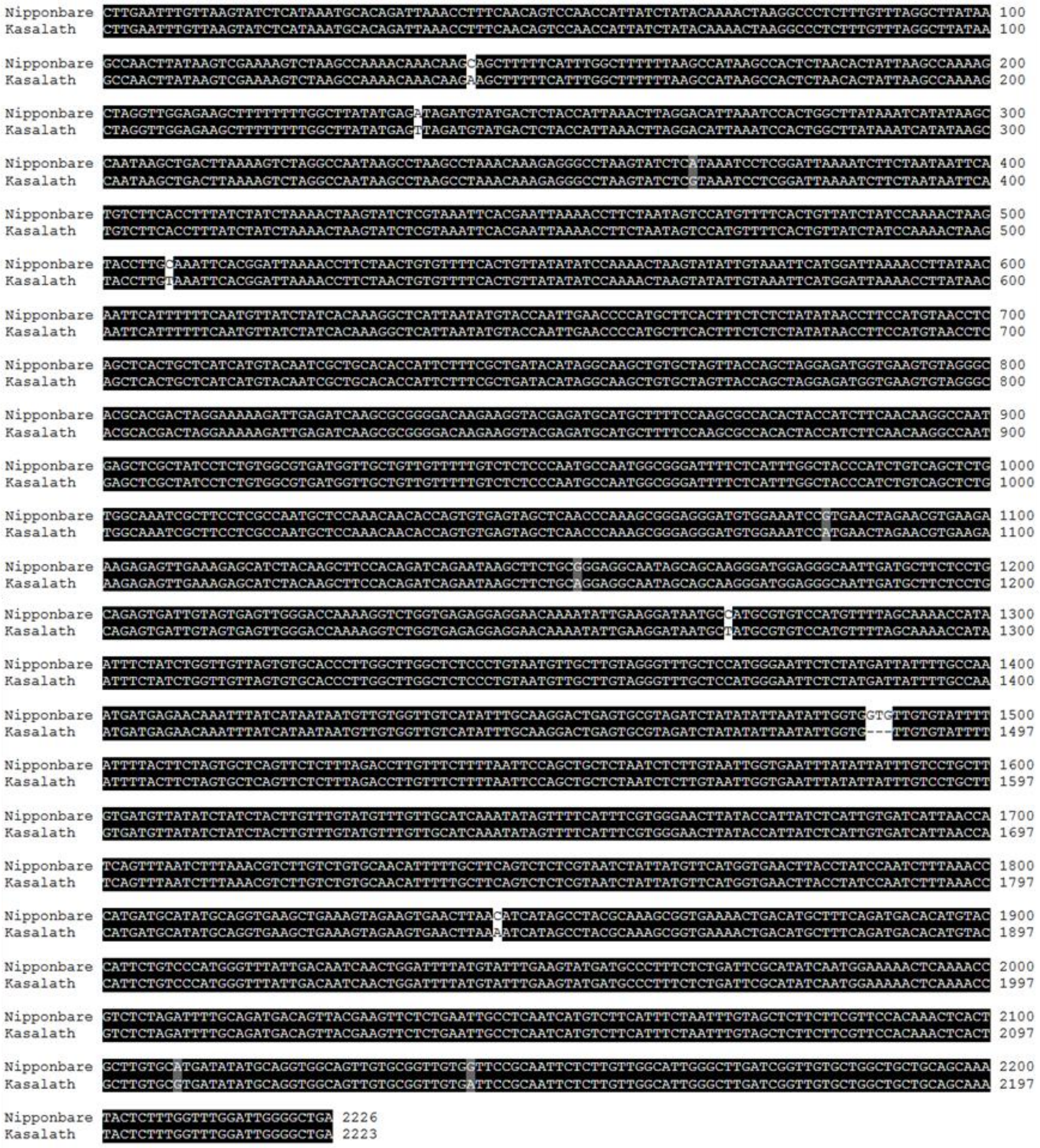

(B)

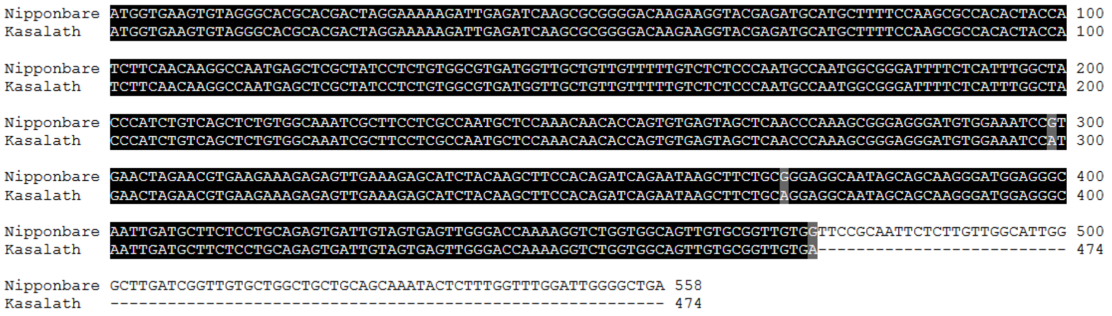

(C)

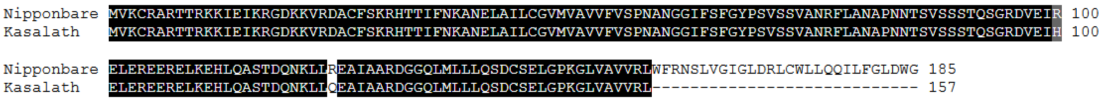

(D)

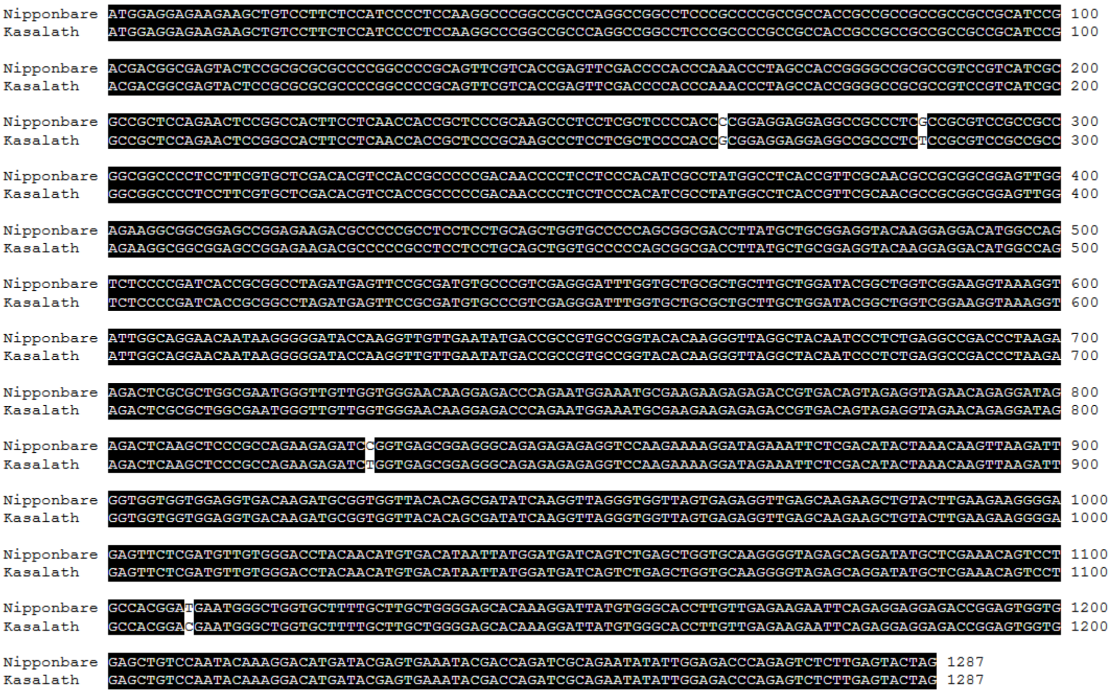

(E)

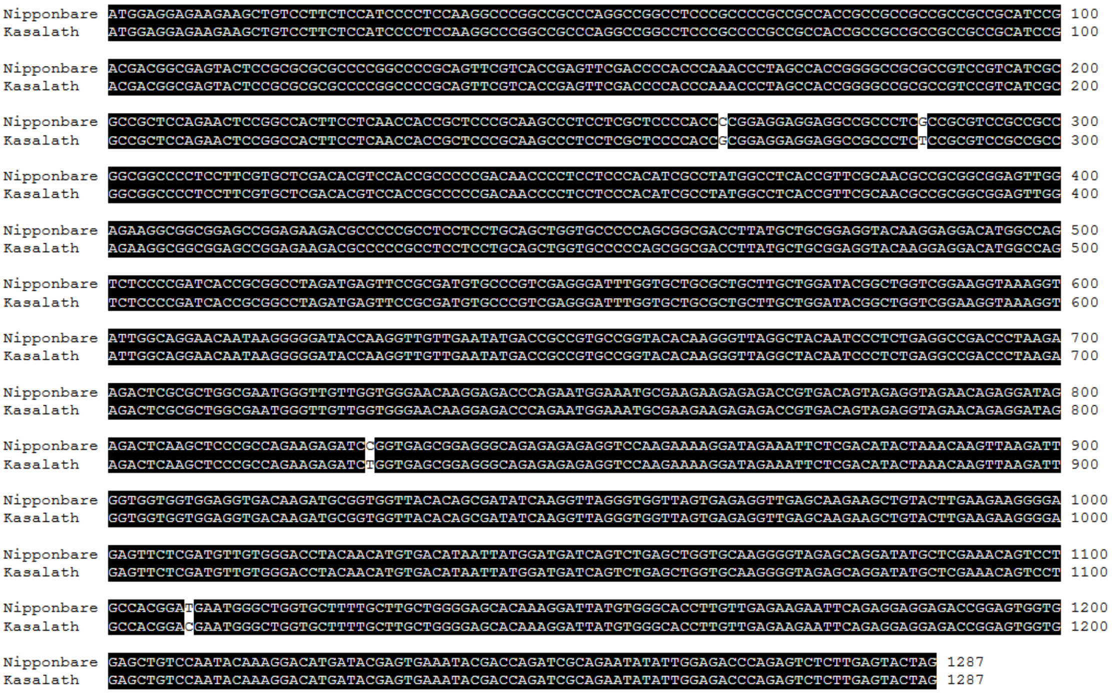

(F)

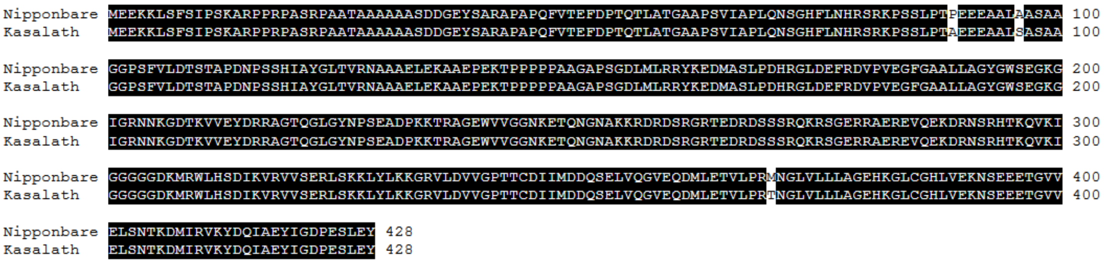

(G)

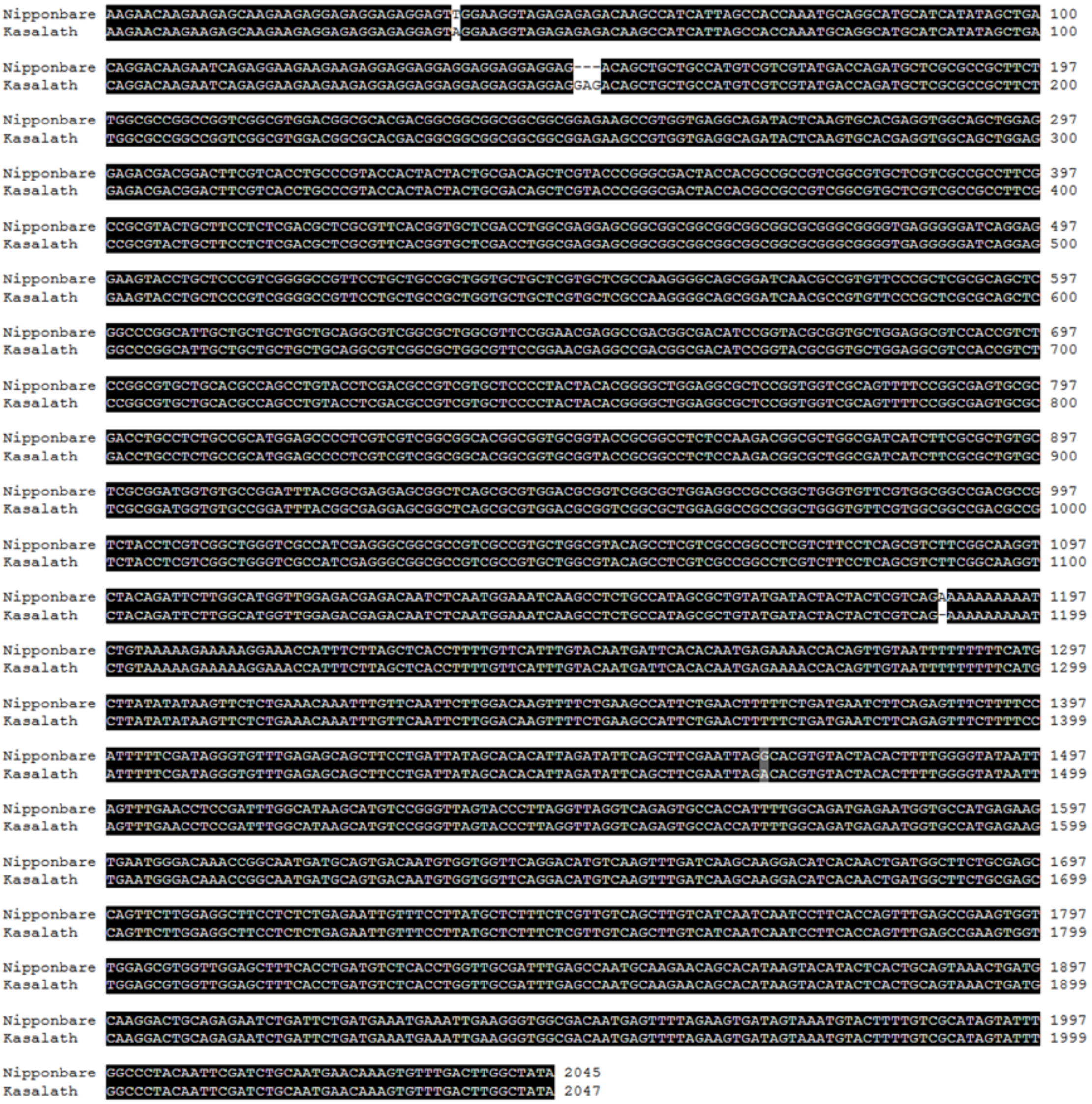

(H)

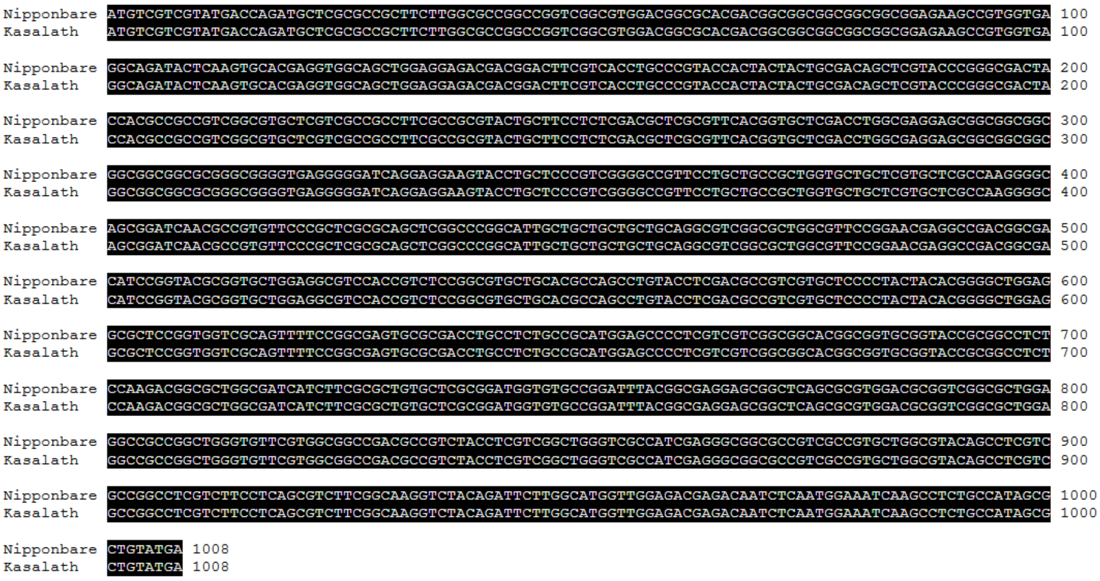

(I)

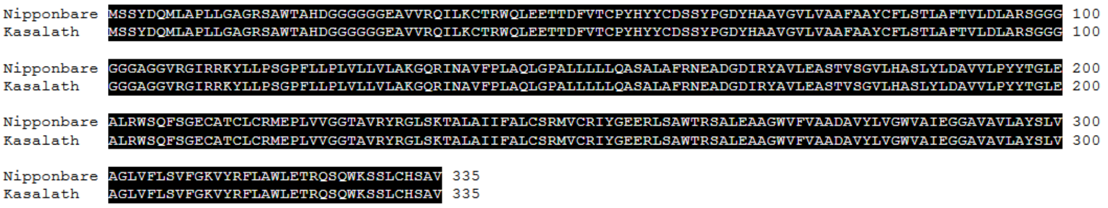

S4 Fig. Alignment of the sequences of three candidate gene in Nipponbare and Kasalath (A)-(C) Gene sequence, coding sequence and deduced amino acid sequence of *LOC_Os03g14850*, respectively. (D)-(F) Gene sequence, coding sequence and deduced amino acid sequence of *LOC_Os03g14860*, respectively. (G)-(I) Gene sequence, coding sequence and deduced amino acid sequence of *LOC_Os03g14880*, respectively.
