# Supplementary material for: Fine Mapping and Candidate Gene Analysis of qSTL3, a Stigma Length-Conditioning Locus in Rice (Oryza sativa L.)
Source: PLoS One. 2015 Jun 1;10(6):e0127938. doi: 10.1371/journal.pone.0127938 (PMC4452489; doi:10.1371/journal.pone.0127938)
Supplement: S6 Fig — M indicates the DNA marker ladder, N and K indicate Nipponbare and Kasalath, No.3-42 indicate the short stigma accessions, and No.43-82 indicate the long stigma accessions. (PDF) [file pone.0127938.s006.pdf]

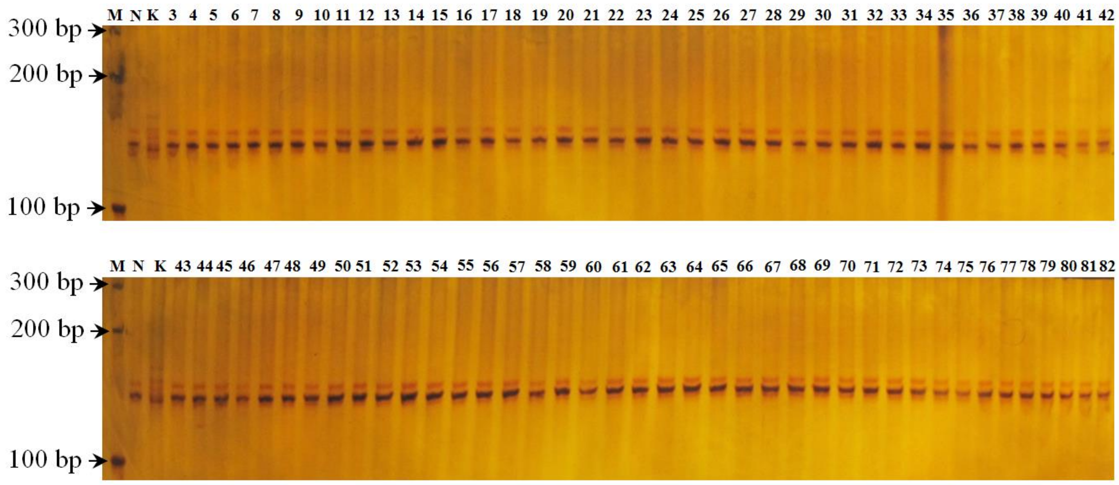

S6 Fig. The profile amplified by the gene-specific marker LQ30 using total DNA of Nipponbare, Kasalath and the 80 accessions with different stigma lengths. M indicates the DNA marker ladder, N and K indicate Nipponbare and Kasalath, No.3-42 indicate the short stigma accessions, and No.43-82 indicate the long stigma accessions.
