# Supplementary material for: Fine Mapping and Candidate Gene Analysis of qSTL3, a Stigma Length-Conditioning Locus in Rice (Oryza sativa L.)
Source: PLoS One. 2015 Jun 1;10(6):e0127938. doi: 10.1371/journal.pone.0127938 (PMC4452489; doi:10.1371/journal.pone.0127938)
Supplement: S1 Table — 1 Both qPES-3 and qSTL3(t) were identified in the BIL population derived from Nipponbare/Kasalath//Nipponbare. 2 All QTLs detected in this reference were not named. We named the QTL for stigma length as qSTL3(t). (PDF) [file pone.0127938.s007.pdf]

S1 Table. Information of QTLs for percentage of exserted stigma and stigma length identified on short arm of chromosome 3.

QTL [1]	Marker interval	LOD	% of phenotypic variance explained	Positive allele	Reference
qPES-3	C563-C63	4.4	16.0	Kasalath	Qiao BJ et al. [8]
qSTL3(t) [2]	C563-C63	3.4	11.8	Kasalath	Uga Y et al. [14]

[1] Both *qPES-3* and *qSTL3*(t) were identified in the BIL population derived from Nipponbare/Kasalath//Nipponbare.

[2] All QTLs detected in this reference did not name. We named the QTL for stigma length as *qSTL3*(t).
