# Supplementary material for: Fine Mapping and Candidate Gene Analysis of qSTL3, a Stigma Length-Conditioning Locus in Rice (Oryza sativa L.)
Source: PLoS One. 2015 Jun 1;10(6):e0127938. doi: 10.1371/journal.pone.0127938 (PMC4452489; doi:10.1371/journal.pone.0127938)
Supplement: S5 Fig — (PDF) [file pone.0127938.s005.pdf]

```
Nipponbare ATGGTGAAGTGTAGGGCACGCACGACTAGGAAAAAGATTGAGATCAAGCGCGGGGACAAGAAGGTACGAGATGCATGCTTTTCCAAGCGCCACACTACCA 100
Zhonghua11 ATGGTGAAGTGTAGGGCACGCACGACTAGGAAAAAGATTGAGATCAAGCGCGGGGACAAGAAGGTACGAGATGCATGCTTTTCCAAGCGCCACACTACCA 100

Nipponbare TCTTCAACAAGGCCAATGAGCTCGCTATCCTCTGTGGCGTGATGGTTGCTGTTGTTTTTGTCTCTCCCAATGCCAATGGCGGATTTTCTCATTTGGCTA 200
Zhonghua11 TCTTCAACAAGGCCAATGAGCTCGCTATCCTCTGTGGCGTGATGGTTGCTGTTGTTTTTGTCTCTCCCAATGCCAATGGCGGATTTTCTCATTTGGCTA 200

Nipponbare CCCATCTGTCAGCTCTGTGGCAAATCGCTTCCTCGCCAATGCTCCAAACAACACCAGTGTGAGTAGCTCAACCCAAAGCGGGAGGGATGTGGAAATCCGT 300
Zhonghua11 CCCATCTGTCAGCTCTGTGGCAAATCGCTTCCTCGCCAATGCTCCAAACAACACCAGTGTGAGTAGCTCAACCCAAAGCGGGAGGGATGTGGAAATCCGT 300

Nipponbare GAACTAGAACGTGAAGAAAGAGAGTTGAAAGAGCATCTACAAGCTTCCACAGATCAGAATAAGCTTCTGCGGGAGGCAATAGCAGCAAGGGATGGAGGGC 400
Zhonghua11 GAACTAGAACGTGAAGAAAGAGAGTTGAAAGAGCATCTACAAGCTTCCACAGATCAGAATAAGCTTCTGCGGGAGGCAATAGCAGCAAGGGATGGAGGGC 400

Nipponbare AATTGATGCTTCTCCTGCAGAGTGATTGTAGTGAGTTGGGACCAAAAGGTCTGGTGAGAGGAGGAACAAAATATTGAAGGATAATGCCATGCGTGTCCAT 500
Zhonghua11 AATTGATGCTTCTCCTGCAGAGTGATTGTAGTGAGTTGGGACCAAAAGGTCTGGTGAGAGGAGGAACAAAATATTGAAGGATAATGCCATGCGTGTCCAT 500

Nipponbare GTTTTAGCAAAACCATAATTTCTATCTGGTTGTTAGTGTGCACCCTTGGCTTGGCTCTCCCTGTAATGTTGCTTGTAGGGTTTGCTCCATGGGAATTCTC 600
Zhonghua11 GTTTTAGCAAAACCATAATTTCTATCTGGTTGTTAGTGTGCACCCTTGGCTTGGCTCTCCCTGTAATGTTGCTTGTAGGGTTTGCTCCATGGGAATTCTC 600

Nipponbare TATGATTATTTTGCCAAATGATGAGAACAAATTTATCATAATAATGTTGTGGTTGTCATATTTGCAAGGACTGAGTGCGTAGATCTATATATTAATATTG 700
Zhonghua11 TATGATTATTTTGCCAAATGATGAGAACAAATTTATCATAATAATGTTGTGGTTGTCATATTTGCAAGGACTGAGTGCGTAGATCTATATATTAATATTG 700

Nipponbare GTGGTGTTGTGTATTTTATTTTACTTCTAGTGCTCAGTTCTCTTTAGACCTTGTTTCTTTTAATTCCAGCTGCTCTAATCTCTTGTAATTGGTGAATTTA 800
Zhonghua11 GTGGTGTTGTGTATTTTATTTTACTTCTAGTGCTCAGTTCTCTTTAGACCTTGTTTCTTTTAATTCCAGCTGCTCTAATCTCTTGTAATTGGTGAATTTA 800

Nipponbare TATTATTTGTCCTGCTTGTGATGTTATATCTATCTACTTGTTTGTATGTTTGTTGCATCAAATATAGTTTTCATTTCGTGGGAACTTATACCATTATCTC 900
Zhonghua11 TATTATTTGTCCTGCTTGTGATGTTATATCTATCTACTTGTTTGTATGTTTGTTGCATCAAATATAGTTTTCATTTCGTGGGAACTTATACCATTATCTC 900

Nipponbare ATTGTGATCATTAACCATCAGTTTAATCTTTAAACGTCTTGTCTGTGCAACATTTTTGCTTCAGTCTCTCGTAATCTATTATGTTCATGGTGAACTTACC 1000
Zhonghua11 ATTGTGATCATTAACCATCAGTTTAATCTTTAAACGTCTTGTCTGTGCAACATTTTTGCTTCAGTCTCTCGTAATCTATTATGTTCATGGTGAACTTACC 1000

Nipponbare TATCCAATCTTTAAACCCATGATGCATATGCAGGTGAAGCTGAAAGTAGAAGTGAACTTAACATCATAGCCTACGCAAAGCGGTGAAAACTGACATGCTT 1100
Zhonghua11 TATCCAATCTTTAAACCCATGATGCATATGCAGGTGAAGCTGAAAGTAGAAGTGAACTTAACATCATAGCCTACGCAAAGCGGTGAAAACTGACATGCTT 1100

Nipponbare TCAGATGACACATGTACCATTCTGTCCCATGGGTTTATTGACAATCAACTGGATTTTATGTATTTGAAGTATGATGCCCTTTCTCTGATTCGCATATCAA 1200
Zhonghua11 TCAGATGACACATGTACCATTCTGTCCCATGGGTTTATTGACAATCAACTGGATTTTATGTATTTGAAGTATGATGCCCTTTCTCTGATTCGCATATCAA 1200

Nipponbare TGGAAAAACTCAAAACCGTCTCTAGATTTTGCAGATGACAGTTACGAAGTTCTCTGAATTGCCTCAATCATGTCTTCATTTCTAATTTGTAGCTCTTCTT 1300
Zhonghua11 TGGAAAAACTCAAAACCGTCTCTAGATTTTGCAGATGACAGTTACGAAGTTCTCTGAATTGCCTCAATCATGTCTTCATTTCTAATTTGTAGCTCTTCTT 1300

Nipponbare CGTTCCACAAACTCACTGCTTGTGCATGATATATGCAGGTGGCAGTTGTGCGGTTGTGGTTCCGCAATTCTCTTGTTGGCATTGGGCTTGATCGGTTGTG 1400
Zhonghua11 CGTTCCACAAACTCACTGCTTGTGCATGATATATGCAGGTGGCAGTTGTGCGGTTGTGGTTCCGCAATTCTCTTGTTGGCATTGGGCTTGATCGGTTGTG 1400

Nipponbare CTGGCTGCTGCAGCAAATACTCTTTGGTTTGGATTGGGGCTGA 1443
Zhonghua11 CTGGCTGCTGCAGCAAATACTCTTTGGTTTGGATTGGGGCTGA 1443
```

S5 Fig. Sequence alignment of *LOC_Os03g14850* beween Nipponbare and Zhonghua11
