# Supplementary material for: Fine Mapping and Candidate Gene Analysis of qSTL3, a Stigma Length-Conditioning Locus in Rice (Oryza sativa L.)
Source: PLoS One. 2015 Jun 1;10(6):e0127938. doi: 10.1371/journal.pone.0127938 (PMC4452489; doi:10.1371/journal.pone.0127938)
Supplement: S2 Table — No.1-2 are the original parents of the fine mapping population, No.3-42 are the accessions with short stigma length, and No.43-82 are the accessions with long stigma length. All stigma length data are given as the mean ± SD (summer 2014, Nanjing, Jiangsu, China). *, ** indicate the least significant difference at a 0.05 and 0.01 probability level compared with Nipponbare by t test, respectively. (PDF) [file pone.0127938.s008.pdf]

S2 Table. Basic information of the accessions used for sequencing analysis.

NO.	Accession name	Origin and geographic coordinates (°E, °N)	mean ±SD
**1**	Nipponbare	Tsukuba, Ibaraki, Japan (36.03, 140.08)	1.72 ±0.02
**2**	Kasalath	Karimganj, Assam, Indian (24.87, 92.35)	1.96 ±0.02**
**3**	Zhongjing 9677	Huaian, Jiangsu, China (118.26, 33.85)	1.63 ±0.01**
**4**	Yanglingdao	Wuxi, Jiangsu, China (121.31, 31.49)	1.64 ±0.02**
**5**	Jia 159	Jiaxing, Zhejiang, China (120.76, 30.75)	1.66 ±0.01**
**6**	Nannongjing 005	Nanjing, Jiangsu, China (118.64, 32.07)	1.66 ±0.02**
**7**	Sanbailitou	Kunshan, Jiangsu, China (120.98, 31.39)	1.67 ±0.01**
**8**	Zhongjing 212	Huaian, Jiangsu, China (118.26, 33.85)	1.67 ±0.02**
**9**	863B	Nanjing, Jiangsu, China (118.64, 32.07)	1.67 ±0.02**
**10**	Cuganhuangdao	Wujiang, Jiangsu, China (120.65, 31.14)	1.68 ±0.02**
**11**	Jianongnuo 2	Jiading, Shanghai, China (121.26, 30.74)	1.68 ±0.02**
**12**	Yangfujing 7	Yangzhou, Jiangsu, China (119.41, 32.39)	1.68 ±0.02**
**13**	Ligengqing	Yixing, Jiangsu, China (119.82, 31.34)	1.69 ±0.02*
**14**	Ebusinuodao	Wuxi, Jiangsu, China (121.31, 31.49)	1.69 ±0.01**
**15**	Zaoshirihuangdao	Wuxian, Jiangsu, China (121.63, 31.26)	1.69 ±0.01**
**16**	Wuyujing 3	Wujin, Jiangsu, China (119.94, 31.70)	1.69 ±0.01**
**17**	Zhendao 99	Zhenjiang, Jiangsu, China (119.25, 31.64)	1.69 ±0.01**
**18**	Yefenghuang	Wujiang, Jiangsu, China (120.65, 31.14)	1.69 ±0.02*
**19**	Ludao	Lianyungang, Jiangsu, China (119.28, 34.58)	1.69 ±0.03*
**20**	Xiangzhunuo	Changshu, Jiangsu, China (120.75, 31.65)	1.70 ±0.01
**21**	Shendao 14	Ji'ning, Shandong, China (116.61, 35.39)	1.70 ±0.01
**22**	Jiangfeng 4	Jiangyin, Jiangsu, China (120.29, 31.92)	1.70 ±0.02
**23**	Nantouzhong	Kunshan, Jiangsu, China (120.98, 31.39)	1.70 ±0.02
**24**	Yaxuenuo	Wuxian, Jiangsu, China (121.63, 31.26)	1.70 ±0.02
**25**	Xiangjing 9407	Ji'ning, Shandong, China (116.61, 35.39)	1.70 ±0.02
**26**	Sihao 4029	Suqian, Jiangsu, China (118.26, 33.85)	1.70 ±0.02
**27**	Guozinuo	Jiading, Shanghai, China (121.26, 30.74)	1.71 ±0.01

**28**	Shiluqing	Kunshan, Jiangsu, China (120.98, 31.39)	1.71±0.01
**29**	Lianjing 9823	Lianyungang, Jiangsu, China (119.28, 34.58)	1.71±0.01
**30**	Jingnuo(zijian)	Nanjing, Jiangsu, China (118.64, 32.07)	1.71±0.02
**31**	5 jing 20	Nanjing, Jiangsu, China (118.64, 32.07)	1.71±0.02
**32**	Xiushui 79	Jiaxing, Zhejiang, China (120.76, 30.75)	1.71±0.02
**33**	Zhongjing 131	Huaian, Jiangsu, China (118.26, 33.85)	1.71±0.02
**34**	Zhenhan 2	Zhenzhou, Henan, China (113.69, 34.79)	1.71±0.02
**35**	Kongqueqing	Kunshan, Jiangsu, China (120.98, 31.39)	1.72±0.01
**36**	Yazihuang	Jinshan, Shanghai, China (121.34, 30.74)	1.72±0.02
**37**	Xudao 5	Xuzhou, Jiangsu, China (117.28, 34.21)	1.72±0.02
**38**	Yangguang 200	Xuzhou, Jiangsu, China (117.28, 34.21)	1.72±0.02
**39**	Shendao 808	Ji'ning, Shandong, China (116.61, 35.39)	1.72±0.02
**40**	Huajing 5	Nanjing, Jiangsu, China (118.64, 32.07)	1.72±0.02
**41**	Yandao8	Yancheng, Jiangsu, China (120.04, 33.37)	1.72±0.02
**42**	H37(6427)	Nanjing, Jiangsu, China (118.64, 32.07)	1.72±0.03
**43**	Huangsandannuo	Wuxian, Jiangsu, China (121.63, 31.26)	1.72±0.02
**44**	Toudengyishixing	Kunshan, Jiangsu, China (120.98, 31.39)	1.72±0.03
**45**	Xiaoqingzhong	Changshu, Jiangsu, China (120.75, 31.65)	1.85±0.01**
**46**	Tieganqing	Wuxian, Jiangsu, China (121.63, 31.26)	1.85±0.02**
**47**	Longgouzhong	Qingpu, Shanghai, China (121.13, 31.15)	1.85±0.02**
**48**	Luohanghuang	Jiangyin, Jiangsu, China (120.29, 31.92)	1.85±0.03**
**49**	5 jing 15	Nanjing, Jiangsu, China (118.64, 32.07)	1.85±0.03**
**50**	Kaiqing	Wuxian, Jiangsu, China (121.63, 31.26)	1.86±0.02**
**51**	Ningjing 2	Nanjing, Jiangsu, China (118.64, 32.07)	1.86±0.02**
**52**	Baikenuo	Wuxian, Jiangsu, China (121.63, 31.26)	1.87±0.01**
**53**	Xiaobaiyedao	Wuxi, Jiangsu, China (121.31, 31.49)	1.88±0.02**
**54**	Zijianxian3	Nanjing, Jiangsu, China (118.64, 32.07)	1.89±0.02**
**55**	Yanjing 9	Yancheng, Jiangsu, China (120.04, 33.37)	1.89±0.02**
**56**	Vietnam-77	Hue, Vietnam (16.46, 107.58)	1.90±0.02**

**57**	Baoxintaihuqing	Wujiang, Jiangsu, China (120.65, 31.14)	1.90±0.03**
**58**	Hongjiaozhan	Guangzhou, Guangdong, China (113.36, 23.15)	1.92±0.02**
**59**	Ⅱ-32B	Changsha, Hunan, China (113.09, 28,20)	1.93±0.03**
**60**	Hongmangshajing	Kunshan, Jiangsu, China (120.98, 31.39)	1.94±0.01**
**61**	Wanyedao	Wuxian, Jiangsu, China (121.63, 31.26)	1.95±0.02**
**62**	Huangsandan	Wujiang, Jiangsu, China (120.65, 31.14)	1.95±0.02**
**63**	Wuxiangjing 14	Wujin, Jiangsu, China (119.94, 31.70)	1.95±0.02**
**64**	Beidao4	Suihua, Heilongjiang, China (127.18, 46.89)	2.01±0.03**
**65**	Wumangzaodao	Changshu, Jiangsu, China (120.75, 31.65)	2.04±0.03**
**66**	Beidao3	Suihua, Heilongjiang, China (127.18, 46.89)	2.07±0.02**
**67**	Vietnam-19	Hue, Vietnam (16.47, 107.58)	2.13±0.02**
**68**	Yuetai B	Guangzhou, Guangdong, China (113.36, 23.15)	2.13±0.04**
**69**	A7444	Changshu, Jiangsu, China (120.75, 31.65)	2.09±0.03
**70**	Tongjing 109	Nantong, Jiangsu, China (120.89, 31.98)	2.15±0.03**
**71**	Yangdao 6	Yangzhou, Jiangsu, China (119.41, 32.39)	2.19±0.02**
**72**	Vietnam-50	Hue, Vietnam (16.47, 107.58)	2.20±0.03**
**73**	H35	Nanjing, Jiangsu, China (118.64, 32.07)	2.22±0.02**
**74**	Vietnam-3	Hue, Vietnam (16.47, 107.58)	2.25±0.02**
**75**	Vietnam-84	Hue, Vietnam (16.47, 107.58)	2.30±0.03**
**76**	Vietnam-9	Hue, Vietnam (16.47, 107.58)	2.34±0.03**
**77**	Huanghuazhan	Guangzhou, Guangdong, China (113.36, 23.15)	2.46±0.03**
**78**	Vietnam-41	Hue, Vietnam (16.47, 107.58)	2.51±0.04**
**79**	Nongxiang 21	Changsha, Hunan, China (113.09, 28,20)	2.60±0.03**
**80**	Xiangwanxian17	Changsha, Hunan, China (113.09, 28,20)	2.64±0.05**
**81**	Nongxiang 26	Changsha, Hunan, China (113.09, 28,20)	2.67±0.04**
**82**	Yuzhenxiang	Changsha, Hunan, China (113.09, 28,20)	2.70±0.03**

No.1-2 are the original parents of the fine mapping population, No.3-42 are the accessions with short stigma length, and No.43-82 are the accessions with long stigma length. All stigma length data are given as the mean ± SD (summer 2014, Nanjing,

Jiangsu, China). *, ** indicate the least significant difference at a 0.05 and 0.01 probability level compared with Nipponbare by *t* test, respectively.
